# Effectiveness and safety of prolonged fenofibrate therapy after the acute phase of hypertriglyceridemic pancreatitis: A long-term retrospective follow-up study

**DOI:** 10.1097/MD.0000000000050647

**Published:** 2026-10-02

**Authors:** Luqiu Zeng, Linling Liu, Li Shen

**Affiliations:** a Gastroenterology Department, Weiyuan County People’s Hospital, Neijiang, Sichuan, China; b Department of Gastroenterology, The Gourong City Fourth People’s Hospital, Zigong, Sichuan, China.

**Keywords:** fenofibrate, hypertriglyceridemic pancreatitis, recurrence, retrospective cohort study, safety, triglyceride control

## Abstract

This study evaluated the association of prolonged fenofibrate therapy with recurrent hypertriglyceridemic pancreatitis (HTGP), long-term triglyceride control, and safety during follow-up. This single-center retrospective cohort study included consecutive patients with HTGP diagnosed between January 2023 and December 2024. Patients who survived the index hospitalization and had post-discharge follow-up data were classified into a prolonged fenofibrate group or a control group according to treatment status. Prolonged fenofibrate therapy was defined as documented continuous use for at least 3 months after discharge. The primary outcome was recurrent HTGP. Secondary outcomes included time to 1st recurrence, descriptive crude recurrence proportions at 6, 12, and 24 months, pancreatitis-related readmission, recurrent hospital stay, follow-up triglyceride (TG) control, and safety outcomes. A total of 200 patients were included, including 107 in the prolonged fenofibrate group and 93 controls. Recurrent HTGP occurred in 17 patients (15.9%) in the fenofibrate group and 24 (25.8%) in the control group. Recurrence was numerically lower with fenofibrate, but the crude comparison was not statistically significant (*P* = .081). Kaplan–Meier analysis showed a similar numerical trend toward better recurrence-free survival (log-rank *P* = .067). In multivariable Cox regression, prolonged fenofibrate therapy showed a potential association with lower recurrence risk, but the estimate was not statistically significant (adjusted hazard ratio 0.58, 95% confidence interval 0.31–1.07, *P* = .082). Previous HTGP remained independently associated with recurrence (adjusted hazard ratio 1.96, 95% confidence interval 1.06–3.63, *P* = .032). Follow-up TG levels were lower in the fenofibrate group (3.9 [2.5–6.2] vs 5.1 [3.0–8.4] mmol/L, *P* = .012), and more patients achieved TG < 5.6 mmol/L (69.2% vs 53.8%, *P* = .026). Safety outcomes were comparable between groups. Prolonged fenofibrate therapy after HTGP was associated with improved long-term triglyceride control and an acceptable safety profile. Recurrence-related outcomes consistently favored fenofibrate numerically, but none reached statistical significance. Larger prospective multicenter studies are needed to clarify any effect on recurrence.

## 1. Introduction

Acute pancreatitis (AP) is one of the most common gastrointestinal disorders requiring emergency hospitalization worldwide and remains a major cause of morbidity and healthcare burden.^[1]^ In recent years, the etiological spectrum of AP has gradually shifted, with hypertriglyceridemia emerging as an increasingly important cause, particularly among patients with obesity, diabetes mellitus, metabolic syndrome, and other lipid-related disorders.^[2]^ Hypertriglyceridemic pancreatitis (HTGP) is generally diagnosed when markedly elevated serum triglyceride (TG) levels are present in the absence of other more likely causes, such as gallstones or excessive alcohol consumption.^[3]^ Clinically, HTGP warrants particular attention because it is frequently associated with pronounced metabolic disturbances, a heightened systemic inflammatory response, and a substantial risk of recurrence following recovery from the initial episode.^[4]^

The pathophysiology of HTGP is closely linked to severe abnormalities in lipid metabolism.^[5]^ When serum TG levels become markedly elevated, excessive triglyceride-rich lipoproteins are hydrolyzed by pancreatic lipase into large amounts of free fatty acids. Once the buffering capacity of albumin is exceeded, these free fatty acids exert direct cytotoxic effects on pancreatic acinar cells and vascular endothelial cells, leading to microcirculatory dysfunction, ischemic injury, oxidative stress, and amplification of local as well as systemic inflammatory cascades. In this context, HTGP may follow a more aggressive clinical course and may predispose affected individuals to recurrent attacks if the underlying metabolic disturbance is not adequately corrected. Although advances in acute-phase management have improved short-term outcomes, prevention of recurrence remains an important yet insufficiently studied aspect of long-term care in patients with HTGP.

Current management of the acute phase of HTGP primarily focuses on supportive care and rapid metabolic stabilization, including fasting, aggressive fluid resuscitation, insulin therapy when indicated, analgesia, and organ support in severe cases.^[5]^ In selected patients, more intensive TG-lowering interventions may also be required. However, successful control of the index episode does not eliminate the risk of future recurrence. Many patients continue to have poorly controlled lipid metabolism after discharge, particularly those with coexisting diabetes, obesity, fatty liver disease, or poor adherence to dietary and pharmacological interventions.^[6]^ Recurrent HTGP may lead to repeated hospitalizations, increased medical costs, and cumulative pancreatic injury, thereby adversely affecting long-term prognosis and quality of life. Accordingly, effective post-acute strategies aimed at sustained TG reduction are essential for secondary prevention.

Long-term control of TG levels after recovery from the acute phase generally relies on a combination of lifestyle modification and pharmacological therapy. Lifestyle interventions, including a low-fat diet, weight reduction, abstinence from alcohol, and optimization of glycemic control, are universally recommended and remain the cornerstone of management. Nevertheless, these measures alone are often insufficient in real-world practice, particularly in patients with severe baseline hypertriglyceridemia or persistent metabolic risk factors.^[7]^ As a result, lipid-lowering medications frequently become necessary to achieve durable TG control and reduce the likelihood of recurrent pancreatitis.^[8]^

Fenofibrate is one of the most commonly prescribed agents for the treatment of hypertriglyceridemia. As a peroxisome proliferator-activated receptor alpha agonist, fenofibrate lowers serum TG levels by enhancing lipoprotein lipase activity, promoting the catabolism of triglyceride-rich lipoproteins, increasing fatty acid oxidation, and reducing hepatic production of very low-density lipoproteins.^[9]^ These pharmacological properties provide a strong biological rationale for its use in the secondary prevention of HTGP. However, despite its widespread use in dyslipidemia management, evidence specifically evaluating the effectiveness of prolonged fenofibrate therapy after the acute phase of HTGP remains limited. In particular, real-world data on its long-term benefit in preventing recurrence are still scarce.

In addition to its potential efficacy, the long-term safety of fenofibrate therapy also merits careful evaluation. Although fenofibrate is generally considered well tolerated, concerns persist regarding possible adverse effects, including elevations in liver enzymes, renal function impairment, and muscle-related toxicity.^[9]^ Such safety considerations may influence clinicians’ prescribing decisions and patients’ long-term adherence, especially in routine clinical settings where close follow-up is not always ensured. At the same time, recent reviews and emerging evidence continue to emphasize that preventing recurrence of HTGP depends on sustained TG reduction after discharge, yet high-quality real-world data remain insufficient.^[10]^ Therefore, assessment of both effectiveness and safety is essential to better define the role of prolonged fenofibrate therapy in patients recovering from HTGP.^[10]^

Given the high recurrence risk of HTGP and the limited real-world evidence regarding long-term pharmacological management, further investigation of sustained fenofibrate therapy is clinically warranted. Therefore, the present study aimed to evaluate the association of prolonged fenofibrate therapy after the acute phase with recurrent HTGP, long-term TG control, and safety in a retrospective follow-up cohort. We hypothesized that prolonged fenofibrate therapy would be associated with better TG control and potentially a lower risk of recurrent HTGP during follow-up.

## 2. Methods

### 2.1. Study design and population

This study was approved by the Ethics Committee of Weiyuan County People’s Hospital. This single-center retrospective cohort study was conducted at Weiyuan County People’s Hospital. Consecutive patients diagnosed with HTGP between January 2023 and December 2024 were screened through the institutional electronic medical record system. Patients who survived the index hospitalization and had available post-discharge follow-up data were considered eligible. According to post-discharge lipid-lowering treatment status, patients were classified into a prolonged fenofibrate therapy group and a control group. The study evaluated recurrence, lipid control, and safety outcomes after the acute phase of HTGP.

### 2.2. Diagnostic criteria

AP was diagnosed when at least 2 of the following 3 criteria were met: characteristic abdominal pain suggestive of AP, serum amylase and/or lipase levels >3 times the upper limit of normal, and imaging findings consistent with AP on ultrasonography, computed tomography, or magnetic resonance imaging. HTGP was diagnosed when AP occurred in the presence of markedly elevated serum TG levels, generally >11.3 mmol/L (1000 mg/dL), or >5.6 mmol/L (500 mg/dL) in the absence of other more likely etiologies such as gallstones, alcohol abuse, drug-induced pancreatitis, or pancreatic malignancy. The severity of AP was classified as mild AP, moderately severe AP, or severe AP according to the revised Atlanta classification.

### 2.3. Inclusion and exclusion criteria

Patients were included if they were aged 18 years or older, had a confirmed diagnosis of HTGP during the index hospitalization, survived the acute episode to hospital discharge, and had available medication and follow-up records after discharge. Patients were excluded if pancreatitis was attributable to another definite etiology, if they had chronic pancreatitis or pancreatic malignancy, if they had severe baseline hepatic or renal dysfunction precluding fenofibrate treatment, or if key clinical data were incomplete or follow-up information was unavailable immediately after discharge.

### 2.4. Exposure definition

The exposure of interest was prolonged fenofibrate therapy after the acute phase. Patients were assigned to the fenofibrate group if fenofibrate was prescribed after discharge and documented as being used continuously for at least 3 months. Treatment continuity was assessed from outpatient prescription records, medication histories recorded at follow-up visits, and other follow-up documentation. In routine practice, fenofibrate was generally administered at a dose of 160 mg once daily or an equivalent approved formulation. Patients who did not receive fenofibrate after discharge or whose records documented discontinuation within 3 months were assigned to the control group. Because this was a retrospective study, no pill counts, medication possession ratios, or systematic pharmacy refill measures were available; therefore, adherence beyond documented treatment continuation could not be quantitatively verified. All patients received routine discharge advice, including dietary modification, weight control, alcohol restriction, and management of metabolic comorbidities as appropriate.

### 2.5. Data collection

Clinical data were extracted from the electronic medical record system and follow-up records using a standardized case report form. The collected variables included age, sex, body mass index (BMI), diabetes mellitus, hypertension, fatty liver disease, previous HTGP, current alcohol use, smoking status, admission TG, total cholesterol, fasting glucose, severity of AP, intensive care unit admission, and length of stay during the index hospitalization. Follow-up variables included recurrent HTGP, time to 1st recurrence, readmission due to pancreatitis, number of readmissions, cumulative recurrent hospital stay, follow-up TG levels, and safety outcomes.

### 2.6. Follow-up and outcome definitions

Patients were followed from the date of hospital discharge after the index episode until recurrence, death, loss to follow-up, or the end of the study period, whichever occurred 1st. Follow-up data were obtained from outpatient visits, inpatient readmission records, and telephone follow-up when necessary. The primary outcome was recurrent HTGP, defined as a new episode of AP after discharge that met the diagnostic criteria for AP and was attributable to hypertriglyceridemia.

The secondary outcomes included time to 1st recurrence, descriptive crude recurrence proportions at 6, 12, and 24 months, pancreatitis-related readmission, number of readmissions per patient, cumulative recurrent hospital stay, and follow-up TG control. Because follow-up duration varied among patients, the percentages reported at 6, 12, and 24 months were treated as descriptive crude cumulative proportions derived from the available follow-up records and did not account for censoring; they were not interpreted as Kaplan–Meier estimates. Accordingly, Kaplan–Meier recurrence-free survival and Cox proportional hazards analyses were used for time-to-event inference. For lipid control assessment, the most recent fasting TG measurement available during follow-up was used. Two prespecified TG control targets were evaluated: TG < 5.6 mmol/L and TG < 2.3 mmol/L. Persistent severe hypertriglyceridemia was defined as follow-up TG remaining ≥11.3 mmol/L.

### 2.7. Safety assessment

Safety outcomes included liver enzyme elevation, creatinine elevation, myalgia, gallstone-related events, severe adverse events, and discontinuation of fenofibrate due to adverse events. Alanine aminotransferase/aspartate aminotransferase (ALT/AST) elevation was defined as alanine aminotransferase or aspartate aminotransferase above the institutional upper limit of normal during follow-up. A stricter alternative, if you want a more publication-style definition, is ALT or AST >2 × upper limit of normal. Creatinine elevation was defined as a documented serum creatinine increase above the upper limit of normal or a clinically relevant increase judged by the treating physician. Myalgia was recorded when muscle pain or discomfort was documented during follow-up without another clear explanation. Drug discontinuation due to adverse events was assessed only in the fenofibrate group.

### 2.8. Statistical analysis

Continuous variables were expressed as mean ± standard deviation for normally distributed data or median (interquartile range) for non-normally distributed data. Categorical variables were presented as number (percentage). Between-group comparisons were performed using the Student *t* test for normally distributed continuous variables, the Mann–Whitney *U* test for skewed continuous variables, and the chi-square test or Fisher exact test for categorical variables, as appropriate. Recurrence-free survival was estimated using the Kaplan–Meier method, and differences between groups were compared using the log-rank test. Cox proportional hazards regression analysis was performed to evaluate factors associated with recurrent HTGP. Prolonged fenofibrate therapy was retained a priori as the primary exposure of interest. The final multivariable model included prolonged fenofibrate therapy, previous HTGP, diabetes mellitus, BMI, and admission TG level. Previous HTGP was included because of its established clinical relevance and its univariate association with recurrence, whereas diabetes mellitus, BMI, and admission TG level were included because of their metabolic relevance and/or a univariate association with recurrence at *P* < .10. Hazard ratios (HRs) and 95% confidence intervals (CIs) were calculated. All tests were 2-sided, and *P* < .05 was considered statistically significant. Statistical analyses were performed using Statistical Package for the Social Sciences version 26.0 (IBM Corp.).

### 2.9. Ethics statement

The study protocol was approved by the Institutional Review Board of Weiyuan County People’s Hospital. Because of the retrospective study design, the requirement for written informed consent was waived in accordance with institutional policy. The study was conducted in accordance with the Declaration of Helsinki.

## 3. Results

### 3.1. Baseline characteristics

A total of 200 patients with hypertriglyceridemic pancreatitis were included in the final analysis, including 107 patients in the prolonged fenofibrate group and 93 in the control group. Baseline characteristics are summarized in Table 1.

**Table 1:** Baseline characteristics of patients with hypertriglyceridemic pancreatitis according to prolonged fenofibrate therapy status.

Variable	Fenofibrate group (n = 107)	Control group (n = 93)	*P* value
Age, yr	42.6 ± 11.4	44.1 ± 12.2	.368
Male sex, n (%)	72 (67.3)	66 (71.0)	.573
BMI, kg/m^2^	28.1 ± 4.1	27.4 ± 3.8	.214
Diabetes mellitus, n (%)	34 (31.8)	34 (36.6)	.475
Hypertension, n (%)	23 (21.5)	24 (25.8)	.474
Fatty liver disease, n (%)	47 (43.9)	45 (48.4)	.525
Previous HTGP, n (%)	27 (25.2)	31 (33.3)	.206
Current alcohol use, n (%)	19 (17.8)	22 (23.7)	.299
Smoking, n (%)	31 (29.0)	33 (35.5)	.325
Admission TG, mmol/L	18.7 (13.9–25.6)	20.1 (14.8–28.4)	.279
Total cholesterol, mmol/L	8.9 ± 2.4	9.2 ± 2.6	.401
Fasting glucose, mmol/L	9.8 ± 3.5	10.4 ± 4.2	.261
Mild AP, n (%)	49 (45.8)	38 (40.9)	.612
Moderately severe AP, n (%)	41 (38.3)	39 (41.9)	
Severe AP, n (%)	17 (15.9)	16 (17.2)	
ICU admission, n (%)	13 (12.1)	15 (16.1)	.417
Length of stay, days	10 (7–14)	11 (8–15)	.171

Values are presented as mean ± standard deviation, median (interquartile range), or number (percentage), as appropriate. *P* values are shown for between-group comparisons.

AP = acute pancreatitis, BMI = body mass index, HTGP = hypertriglyceridemic pancreatitis, ICU = intensive care unit, TG = triglyceride.

The 2 groups were generally comparable at baseline. Mean age was 42.6 ± 11.4 years in the fenofibrate group and 44.1 ± 12.2 years in the control group (*P* = .368), and the proportion of male patients was similar between groups (67.3% vs 71.0%, *P* = .573). No significant between-group differences were observed in BMI, diabetes mellitus, hypertension, fatty liver disease, previous HTGP, current alcohol use, or smoking status (all *P* > .05). Admission triglyceride levels were also comparable (18.7 [13.9–25.6] vs 20.1 [14.8–28.4] mmol/L, *P* = .279), as were total cholesterol and fasting glucose levels. The distributions of acute pancreatitis severity, intensive care unit admission, and length of stay during the index hospitalization did not significantly differ between groups.

### 3.2. Recurrence outcomes

Recurrence outcomes during follow-up are presented in Table 2. Median follow-up duration was 12.5 (8.0–17.0) months in the fenofibrate group and 11.0 (7.0–16.0) months in the control group (*P* = .287). Because individual follow-up duration varied, the fixed-time recurrence percentages below are presented descriptively and should not be interpreted as censoring-adjusted estimates.

**Table 2:** Recurrence outcomes during follow-up.

Outcome	Fenofibrate group (n = 107)	Control group (n = 93)	*P* value
Median follow-up, mo	12.5 (8.0–17.0)	11.0 (7.0–16.0)	.287
Recurrent HTGP, n (%)	17 (15.9)	24 (25.8)	.081
Time to 1st recurrence, mo	8.0 (5.0–13.0)	6.0 (3.0–10.0)	.094
6-mo recurrence, n (%)	6 (5.6)	10 (10.8)	.177
12-mo recurrence, n (%)	13 (12.1)	20 (21.5)	.072
24-mo recurrence, n (%)	17 (15.9)	24 (25.8)	.081
Pancreatitis-related readmission, n (%)	19 (17.8)	27 (29.0)	.061
Number of readmissions per patient	0.24 ± 0.61	0.39 ± 0.81	.128
Total recurrent hospital stay, d	3 (0–7)	4 (0–10)	.117
Kaplan–Meier analysis	log-rank *P* = .067		

HTGP = hypertriglyceridemic pancreatitis.

During follow-up, recurrent HTGP occurred in 17 patients (15.9%) in the fenofibrate group and 24 patients (25.8%) in the control group. Although recurrence was numerically lower in the fenofibrate group, the difference did not reach statistical significance (*P* = .081). Median time to 1st recurrence was 8.0 (5.0–13.0) months in the fenofibrate group and 6.0 (3.0–10.0) months in the control group (*P* = .094).

At the prespecified landmarks, the descriptive crude cumulative recurrence proportions were numerically lower in the fenofibrate group at 6 months (5.6% vs 10.8%, *P* = .177), 12 months (12.1% vs 21.5%, *P* = .072), and 24 months (15.9% vs 25.8%, *P* = .081). These crude landmark percentages do not account for variable follow-up or censoring and are therefore supplementary to the Kaplan–Meier analysis. Similarly, pancreatitis-related readmission occurred numerically less frequently in the fenofibrate group than in the control group (17.8% vs 29.0%, *P* = .061), but this difference was not statistically significant. The mean number of readmissions per patient and total recurrent hospital stay also did not significantly differ between groups (*P* = .128 and .117, respectively).

Kaplan–Meier analysis showed a numerical trend toward better recurrence-free survival in the fenofibrate group, but the difference was not statistically significant (log-rank *P* = .067).

### 3.3. Cox regression analysis for recurrence

Cox regression analyses are shown in Table 3. In univariate analysis, prolonged fenofibrate therapy showed a numerical association with lower recurrence risk, but the association was not statistically significant (HR 0.63, 95% CI 0.34–1.15, *P* = .132). Previous HTGP was significantly associated with recurrence (HR 2.18, 95% CI 1.21–3.95, *P* = .010), while diabetes mellitus, BMI, and admission TG level met the prespecified criterion for consideration in the multivariable model (*P* < .10) and were also clinically relevant metabolic covariates.

**Table 3:** Cox regression analysis for recurrence of hypertriglyceridemic pancreatitis.

Variable	Univariate HR (95% CI)	*P* value	Multivariable HR (95% CI)	*P* value
Prolonged fenofibrate therapy	0.63 (0.34–1.15)	.132	0.58 (0.31–1.07)	.082
Age, per yr	1.01 (0.98–1.03)	.612	–	–
Male sex	1.16 (0.62–2.15)	.644	–	–
BMI, per kg/m^2^	1.06 (0.99–1.14)	.091	1.04 (0.97–1.12)	.246
Diabetes mellitus	1.71 (0.95–3.09)	.074	1.48 (0.80–2.75)	.211
Previous HTGP	2.18 (1.21–3.95)	.010	1.96 (1.06–3.63)	.032
Admission TG, per 1 mmol/L	1.03 (1.00–1.06)	.058	1.02 (0.99–1.05)	.144
Severe AP	1.41 (0.71–2.82)	.328	–	–

AP = acute pancreatitis, BMI = body mass index, CI = confidence interval, HR = hazard ratio, HTGP = hypertriglyceridemic pancreatitis, TG = triglyceride.

After multivariable adjustment for prolonged fenofibrate therapy, previous HTGP, diabetes mellitus, BMI, and admission TG level, the point estimate for prolonged fenofibrate therapy remained below 1, but the association with recurrence was not statistically significant (adjusted HR 0.58, 95% CI 0.31–1.07, *P* = .082). Previous HTGP remained independently associated with recurrence (adjusted HR 1.96, 95% CI 1.06–3.63, *P* = .032). The other covariates in the adjusted model were not independently associated with recurrence.

### 3.4. Follow-up triglyceride control

Follow-up lipid outcomes are summarized in Table 4. Patients receiving prolonged fenofibrate therapy had significantly lower follow-up TG levels than controls (3.9 [2.5–6.2] vs 5.1 [3.0–8.4] mmol/L, *P* = .012). In addition, the proportion of patients achieving TG < 5.6 mmol/L was significantly higher in the fenofibrate group than in the control group (69.2% vs 53.8%, *P* = .026).

**Table 4:** Follow-up triglyceride control.

Variable	Fenofibrate group (n = 107)	Control group (n = 93)	*P* value
Follow-up TG, mmol/L	3.9 (2.5–6.2)	5.1 (3.0–8.4)	.012
TG < 5.6 mmol/L achieved, n (%)	74 (69.2)	50 (53.8)	.026
TG < 2.3 mmol/L achieved, n (%)	23 (21.5)	14 (15.1)	.243
Persistent severe hypertriglyceridemia, n (%)	14 (13.1)	22 (23.7)	.054

TG = triglyceride.

By contrast, the proportion achieving TG < 2.3 mmol/L did not significantly differ between groups (21.5% vs 15.1%, *P* = .243). Persistent severe hypertriglyceridemia was less frequent in the fenofibrate group than in the control group (13.1% vs 23.7%), with borderline statistical significance (*P* = .054).

### 3.5. Safety outcomes

Safety outcomes are shown in Table 5. ALT/AST elevation, defined as a value above the institutional upper limit of normal, occurred in 10 patients (9.3%) in the fenofibrate group and 7 patients (7.5%) in the control group (*P* = .647). Creatinine elevation, defined as a value above the institutional upper limit of normal, was observed in 7 patients (6.5%) and 4 patients (4.3%), respectively (*P* = .497). Myalgia was reported in 5 patients (4.7%) in the fenofibrate group and 2 patients (2.2%) in the control group (*P* = .453).

**Table 5:** Safety outcomes during follow-up.

Safety outcome	Fenofibrate group (n = 107)	Control group (n = 93)	*P* value
ALT/AST elevation, n (%)	10 (9.3)	7 (7.5)	.647
Creatinine elevation, n (%)	7 (6.5)	4 (4.3)	.497
Myalgia, n (%)	5 (4.7)	2 (2.2)	.453
Gallstone-related events, n (%)	2 (1.9)	3 (3.2)	.667
Drug discontinuation due to adverse events, n (%)	6 (5.6)	–	–
Severe adverse events, n (%)	1 (0.9)	2 (2.2)	0.601

ALT = alanine aminotransferase, AST = aspartate aminotransferase.

Gallstone-related events were uncommon and comparable between groups (1.9% vs 3.2%, *P* = .667). Drug discontinuation due to adverse events occurred in 6 patients (5.6%) in the fenofibrate group. Severe adverse events were rare overall, with no significant between-group difference (0.9% vs 2.2%, *P* = .601).

Overall, prolonged fenofibrate therapy was associated with significantly better TG control during follow-up and showed an acceptable safety profile. Recurrence outcomes showed a numerical trend favoring the fenofibrate group, but the association with lower recurrence was not statistically significant in this cohort.

## 4. Discussion

In this single-center retrospective cohort study, prolonged fenofibrate therapy after the acute phase of hypertriglyceridemic pancreatitis was associated with significantly better long-term triglyceride control. Recurrence rates, time to 1st recurrence, pancreatitis-related readmission, and recurrence-free survival showed numerical trends favoring the fenofibrate group, but none of the crude recurrence comparison, log-rank test, or adjusted Cox estimate reached conventional statistical significance. Fenofibrate was generally well tolerated, with no significant increase in major adverse events. Previous HTGP remained independently associated with recurrence after multivariable adjustment. Taken together, the most robust finding of this study is the improvement in long-term TG control, whereas the recurrence findings should be interpreted as a potential association that requires confirmation rather than evidence of a preventive effect.

Recurrence remains an important challenge in the long-term management of HTGP. Previous studies have shown that recurrence after an initial episode of HTGP is common and closely related to post-discharge lipid control. A systematic review and meta-analysis reported that recurrence after a 1st episode of acute pancreatitis is frequent overall, with hypertriglyceridemia among the etiologies associated with relatively high pooled recurrence rates.^[11–14]^ In addition, prospective cohort studies have shown that recurrence after 1st-episode HTGP remains common and is associated with higher post-index TG and glucose levels, whereas long-term TG-lowering treatment may be associated with lower recurrence risk.^[15–19]^ In our cohort, the approximately 10-percentage-point crude difference in recurrence represents a numerical signal only, because the between-group comparison and time-to-event analyses were not statistically significant. Therefore, this observation should be considered hypothesis-generating rather than evidence of recurrence prevention.

A particularly important finding of the present study was the significant improvement in long-term TG control among patients treated with fenofibrate. This observation is biologically plausible and consistent with current understanding of triglyceride metabolism and HTGP pathogenesis. Recent narrative and mechanistic reviews have emphasized that sustained TG reduction after discharge remains a cornerstone of secondary management in HTGP.^[20]^ Prospective real-world data also suggest that triglyceride-lowering strategies are closely linked to metabolic improvement in HTG-AP.^[21]^ Moreover, ambulatory TG levels during follow-up have been shown to correlate with the risk of recurrent pancreatitis, reinforcing the importance of long-term metabolic monitoring rather than relying solely on TG values at the index admission.^[22]^ The fact that lipid control improved significantly in our study, whereas recurrence reduction remained statistically inconclusive, likely reflects the multifactorial nature of recurrence. In clinical practice, recurrent episodes are influenced not only by lipid-lowering treatment but also by dietary adherence, alcohol intake, glycemic control, obesity, and long-term treatment compliance, all of which are difficult to capture precisely in retrospective cohorts.^[23]^

Another notable finding was that previous HTGP remained independently associated with recurrence after multivariable adjustment. This result is consistent with the broader recurrence literature suggesting that prior disease history identifies patients with persistently unstable metabolic risk and a greater likelihood of repeated attacks.^[24]^ Other studies have further suggested that recurrence risk may be influenced by specific biological susceptibility, including abnormalities in lipid metabolism pathways and, in selected patients, immunologic or genetic mechanisms. Clinically, this implies that patients with previous HTGP should be considered a higher-risk subgroup and may benefit from closer lipid monitoring, stricter follow-up, and earlier intensification of long-term preventive strategies. By contrast, the absence of independent associations for diabetes mellitus, BMI, and admission TG level in the adjusted model may reflect limited statistical power, collinearity with longitudinal metabolic control, or the possibility that recurrence risk is shaped more by post-discharge metabolic exposure than by baseline characteristics alone.

The safety findings of this study are also clinically relevant. Although elevations in liver enzymes and creatinine, and reports of myalgia, were numerically more frequent in the fenofibrate group, these differences were not statistically significant, and severe adverse events were rare. This overall pattern is consistent with current evidence indicating that fenofibrate is generally tolerable when prescribed appropriately and accompanied by routine monitoring. Several limitations should nevertheless be acknowledged. The retrospective, single-center design introduces potential selection bias and limits generalizability. Treatment allocation was not randomized, and residual confounding cannot be excluded. Furthermore, actual medication adherence could not be quantified using pill counts, medication possession ratios, or systematic pharmacy refill data, and dietary compliance, alcohol abstinence, and lifestyle modification could not be fully quantified. The relatively small number of recurrent events likely reduced statistical power. In addition, the descriptive recurrence proportions at fixed time points did not account for censoring and should be interpreted alongside the Kaplan–Meier and Cox analyses. Despite these limitations, our study provides real-world evidence that prolonged fenofibrate therapy after HTGP is associated with better long-term lipid control without a clear increase in major safety events, while the observed numerical trend toward lower recurrence remains hypothesis-generating.

## 5. Conclusion

In this retrospective follow-up cohort, prolonged fenofibrate therapy after the acute phase of hypertriglyceridemic pancreatitis was associated with significantly improved long-term triglyceride control and an acceptable safety profile. Recurrence rates, pancreatitis-related readmission, and recurrence-free survival showed numerical trends favoring the fenofibrate group, but the association between prolonged fenofibrate therapy and recurrence was not statistically significant. Previous HTGP remained independently associated with recurrence, suggesting that patients with prior episodes may represent a high-risk subgroup requiring closer long-term surveillance. Overall, these findings support a potential role for prolonged fenofibrate therapy in post-acute metabolic management after HTGP, while any effect on recurrence prevention requires confirmation in larger prospective multicenter studies.

## Author contributions

**Conceptualization:** Luqiu Zeng, Linling Liu, Li Shen.

**Data curation:** Luqiu Zeng, Linling Liu, Li Shen.

**Formal analysis:** Luqiu Zeng, Linling Liu, Li Shen.

**Funding acquisition:** Luqiu Zeng.

**Investigation:** Luqiu Zeng.

**Writing – original draft:** Luqiu Zeng.

**Writing – review & editing:** Luqiu Zeng.

## References

[1] Tenner S Vege SS Sheth SG. American college of gastroenterology guidelines: management of acute pancreatitis. Am J Gastroenterol. 2024;119:419–37. 10.14309/ajg.0000000000002645 PMC13221274 38857482

[2] Rawla P Sunkara T Thandra KC Gaduputi V. Hypertriglyceridemia-induced pancreatitis: updated review of current treatment and preventive strategies. Clin J Gastroenterol. 2018;11:441–8. 10.1007/s12328-018-0881-1 29923163

[3] Yang AL McNabb-Baltar J. Hypertriglyceridemia and acute pancreatitis. Pancreatology. 2020;20:795–800. 10.1016/j.pan.2020.06.005 32571534

[4] Ding L Li S Cao L. Recurrence of hypertriglyceridemia-associated acute pancreatitis: a multicenter, prospective cohort study. Eur J Intern Med. 2024;125:98–103. 10.1016/j.ejim.2024.03.022 38538416

[5] Hernandez P Passi N Modarressi T. Clinical management of hypertriglyceridemia in the prevention of cardiovascular disease and pancreatitis. Curr Atheroscler Rep. 2021;23:72. 10.1007/s11883-021-00962-z PMC8436578 34515873

[6] Virani SS Morris PB Agarwala A. 2021 ACC expert consensus decision pathway on the management of ASCVD risk reduction in patients with persistent hypertriglyceridemia: a report of the American College of Cardiology Solution Set Oversight Committee. J Am Coll Cardiol. 2021;78:960–93. 10.1016/j.jacc.2021.06.011 34332805

[7] Ezhov MV Arutyunov GP. Effectiveness and safety of fenofibrate in routine treatment of patients with hypertriglyceridemia and metabolic syndrome. Diseases. 2023;11:140. 10.3390/diseases11040140 PMC10594425 37873784

[8] MacDonald BJ Garrison SR Turgeon RD. Reducing pancreatitis risk in patients with hypertriglyceridemia. Can Fam Physician. 2023;69:849. 10.46747/cfp.6912849 PMC10949260 38092449

[9] Subramanian S Soran H Kessler A S McClain MR Eckel RH Rosenson RS. Prevention and treatment of hypertriglyceridemia-mediated acute pancreatitis: a narrative review. Eur J Intern Med. 2026;145:106648. 10.1016/j.ejim.2025.106648 41444050

[10] Lu J Wang Z Mei W. A systematic review of the epidemiology and risk factors for severity and recurrence of hypertriglyceridemia-induced acute pancreatitis. BMC Gastroenterol. 2025;25:374. 10.1186/s12876-025-03954-4 PMC12082898 40375154

[11] Li S Gao L Gong H. Recurrence rates and risk factors for recurrence after first episode of acute pancreatitis: a systematic review and meta-analysis. Eur J Intern Med. 2023;114:47–54. 10.1016/j.ejim.2023.06.006 37330318

[12] Gao L Li W. Hypertriglyceridemia and acute pancreatitis: clinical and basic research – a narrative review. J Pancreatol. 2023;7:53–60.

[13] Zhou J Wang Z Liu Q. Triglyceride-lowering therapies in hypertriglyceridemia-associated acute pancreatitis in China: a multicentre prospective cohort study. BMC Med. 2024;22:535. 10.1186/s12916-024-03755-8 PMC11566486 39548430

[14] Hansen SEJ Varbo A Nordestgaard BG Langsted A. Hypertriglyceridemia-associated pancreatitis: new concepts and potential mechanisms. Clin Chem. 2023;69:1132–44. 10.1093/clinchem/hvad094 37530032

[15] Ding L Guan L Li X. Recurrence for patients with first episode of hypertriglyceridemia-induced acute pancreatitis: a prospective cohort study. J Clin Lipidol. 2023;17:94–102. 10.1016/j.jacl.2022.11.006 36697323

[16] Zhang G Yang Q Mao W. GPIHBP1 autoantibody is an independent risk factor for the recurrence of hypertriglyceridemia-induced acute pancreatitis. J Clin Lipidol. 2022;16:626–34. 10.1016/j.jacl.2022.08.001 36064883

[17] Wu BU Batech M Dong EY Duan L Yadav D Chen W. Influence of ambulatory triglyceride levels on risk of recurrence in patients with hypertriglyceridemic pancreatitis. Dig Dis Sci. 2019;64:890–7. 10.1007/s10620-018-5226-x 30094622

[18] Zafrir B Saliba W Jubran A Hijazi R Shapira C. Severe hypertriglyceridemia-related pancreatitis: characteristics and predictors of recurrence. Pancreas. 2019;48:182–6. 10.1097/MPA.0000000000001235 30629026

[19] Guan L Ding L Li X. Serum triglyceride levels are associated with recurrence in patients with acute hypertriglyceridemic pancreatitis. Front Med (Lausanne). 2023;10:1079637. 10.3389/fmed.2023.1079637 PMC10050706 37007797

[20] Hansen SEJ Madsen CM Varbo A Nordestgaard BG Langsted A. Genetic variants associated with increased plasma levels of triglycerides, via effects on the lipoprotein lipase pathway, increase risk of acute pancreatitis. Clin Gastroenterol Hepatol. 2021;19:1652–61.e6. 10.1016/j.cgh.2020.08.016 32801009

[21] Simha V. Management of hypertriglyceridemia. BMJ. 2020;371:m3109. 10.1136/bmj.m3109 33046451

[22] Edu AV Apostol AM Simion M Podda M Bacalbasa N Badiu DC. Hypertriglyceridemia-induced acute pancreatitis – the current state of knowledge. Diagnostics (Basel). 2024;14:1105.

[23] Piplani S Jain A Singh K. Efficacy and adverse effects of insulin versus plasmapheresis in patients with hypertriglyceridemia-3-induced acute pancreatitis: a systematic review and meta-analysis. Ann Gastroenterol. 2024;37:109–16. 10.20524/aog.2023.0849 PMC10785016 38223249

[24] Tang L Jia Q Liu N. Lipid metabolism for predicting the recurrence of hypertriglyceridemic acute pancreatitis. Heliyon. 2023;9:e17443. 10.1016/j.heliyon.2023.e17443 PMC10333607 37441413

